# Surgical Implications of the Arterial Anatomy around the Knee: A Cadaveric Pictorial Essay

**DOI:** 10.3390/diagnostics11112004

**Published:** 2021-10-28

**Authors:** Apostolos Fyllos, Vasileios Raoulis, Vasileios Mitrousias, Konstantinos Banios, Dimitrios Chytas, Aristeidis Zibis

**Affiliations:** 1 Laboratory of Anatomy, Department of Medicine, School of Health Sciences, University of Thessaly, 3 University Str, Biopolis, 41110 Larissa, Greece; apofyl@hotmail.com (A.F.); v_raoulis@yahoo.gr (V.R.); vasileiosmitrousias@gmail.com (V.M.); konmpanios@hotmail.com (K.B.); 2 Department of Orthopedic Surgery, University Hospital of Larissa, 3 University Str, Biopolis, 41110 Larissa, Greece; 3 Department of Orthopedic Surgery, General Hospital of Karditsa, Peripheral Road Karditsa-Kastania, 43100 Karditsa, Greece; 4 Department of Physiotherapy, University of Peloponnese, 20 Plateon Str, 23100 Sparta, Greece; dimitrioschytas@gmail.com

**Keywords:** clinical anatomy, surgical anatomy, radiological anatomy, effectiveness in teaching anatomy, surgical anatomical variations, advanced dissection techniques, anatomical implications of clinical problems

## Abstract

We completed an anatomic cadaver study in order to examine the arterial supply around the knee and to create useful images regarding the arterial surgical anatomy around the knee. A total of four unmatched fresh-frozen cadaveric knees were utilized. There was no medical history of osteoporosis, bony or soft-tissue injury or surgery in any of the knees. The femoral arteries were cannulated with a large catheter at the proximal aspect of the cadavers, and liquid latex in different colours was injected. Τhe specimens were then placed into a bath of 8.0% sodium hypochlorite to complete debridement of the soft tissues to various degrees. The specimens were checked every 15 to 30 min until adequate debridement occurred, and photographs were taken during each stage of this process. Sodium hypochlorite, among others, chemically debrides the vessel walls leaving the casts of the vessel lumens filled with solid coloured latex in order to illustrate the vascular supply pattern to the structures of interest. Knowing the probability of where these arteries should be located adds to the knowledge that surgeons have at their disposal when performing procedures involving arthroscopy, arthroplasty and osteotomies, which can help decrease unnecessary damage to the arteries.

**Figure 1:**
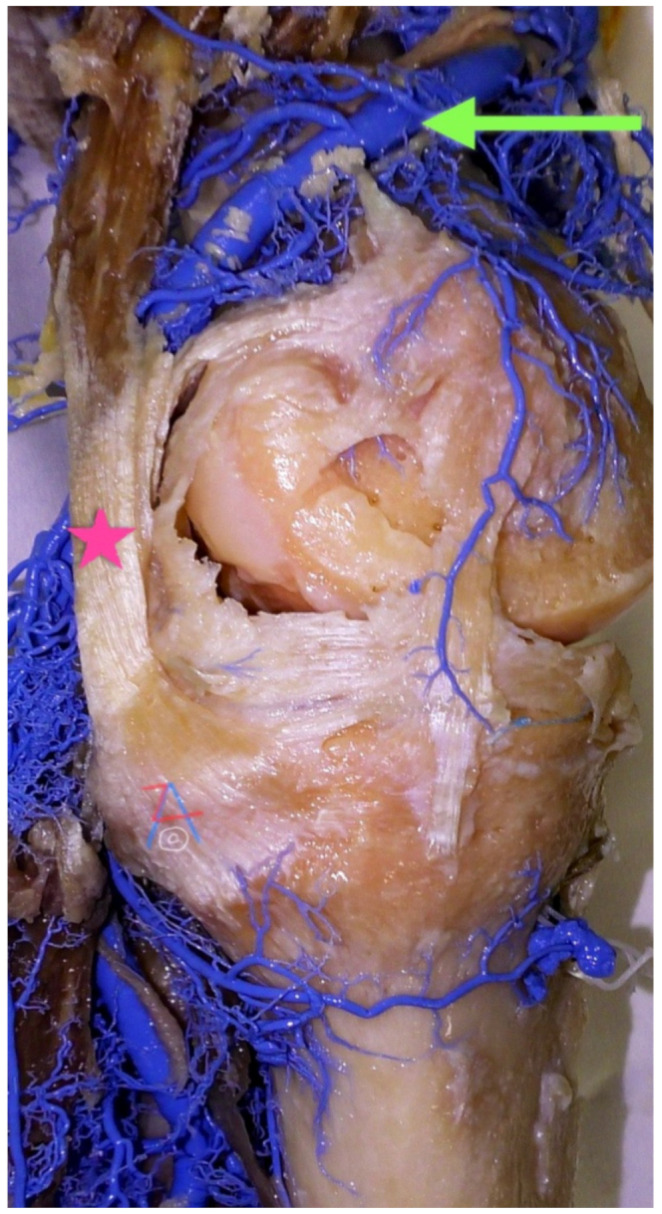
Left knee, medial side. The transition of the femoral artery (green arrow) through the distal part of the Hunter’s canal to become the popliteal artery posteriorly. Pink star: the remains of hamstrings after chemical debridement before forming pes anserinus.

**Figure 2:**
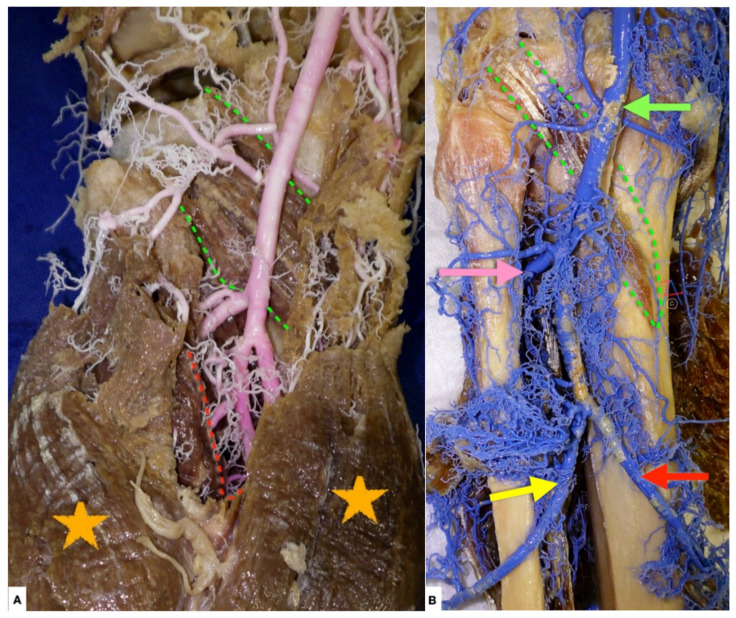
(**A**) Left knee, posterior view. The popliteal artery (pink vessel) is the continuation of the femoral artery, and it exits from Hunter canal and enters the popliteal fossa at the junction of the middle and lower third of the femur. Distally, the artery runs superficially to the popliteus muscle fascia (green dotted line) In the popliteal fossa, the artery is deep and medial to the popliteal vein and tibial nerve. Proximally, it is separated from the femoral bone by a thick pad of fat. There is firm anatomic fixation of the popliteal artery in the adductor hiatus tendineus proximally and the arcus tendineus musculi solei distally (red dotted line). Orange stars: medial and lateral head of gastrocnemius muscle. (**B**) Left knee, posterior view, all muscles and soft tissues chemically debrided. The popliteal artery (green arrow) ends at the lower border of the popliteus (green dotted line) by dividing into the anterior (pink arrow) and posterior tibial (red arrow) and peroneal (yellow arrow) arteries. The calculated incidence of popliteal artery injury is 5.67 per 10,000 cases of TKA, a percentage that rises in revision TKA cases [1]. Approximately 10% of patients with knee dislocations have popliteal artery injury [2].

**Figure 3:**
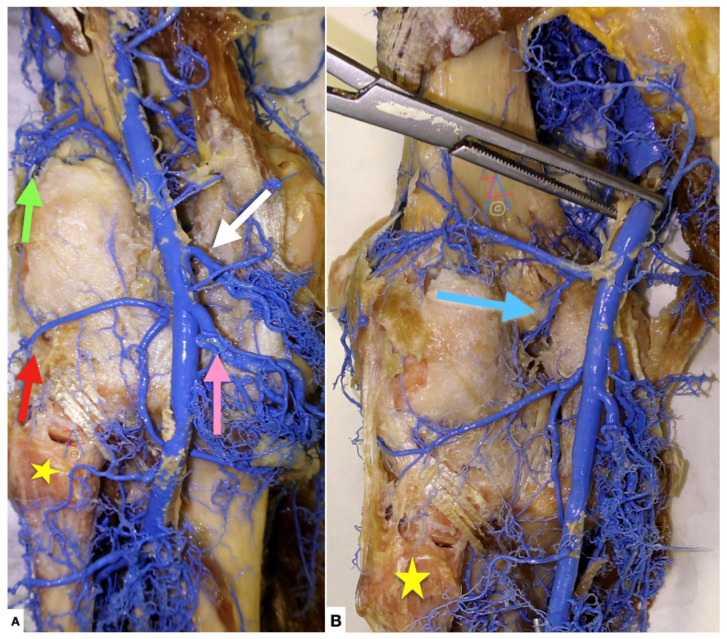
(**A**) Left knee, posterior view. The popliteal artery gives off numerous muscular branches and five genicular branches. They participate in the formation of the periarticular genicular anastomosis, which provides collateral circulation capable of maintaining blood supply to the leg during full knee flexion, which may kink the popliteal artery. Five genicular branches of the popliteal artery supply the capsule and ligaments of the knee joint: The superior lateral genicular artery (green arrow), the superior medial genicular artery (white arrow), the middle genicular artery, the inferior lateral genicular artery (red arrow) and the inferior medial genicular artery (pink arrow). Yellow star: head of fibula. (**B**) Same specimen as in (**A**), where the middle genicular artery (light blue arrow) is discernible.

**Figure 4:**
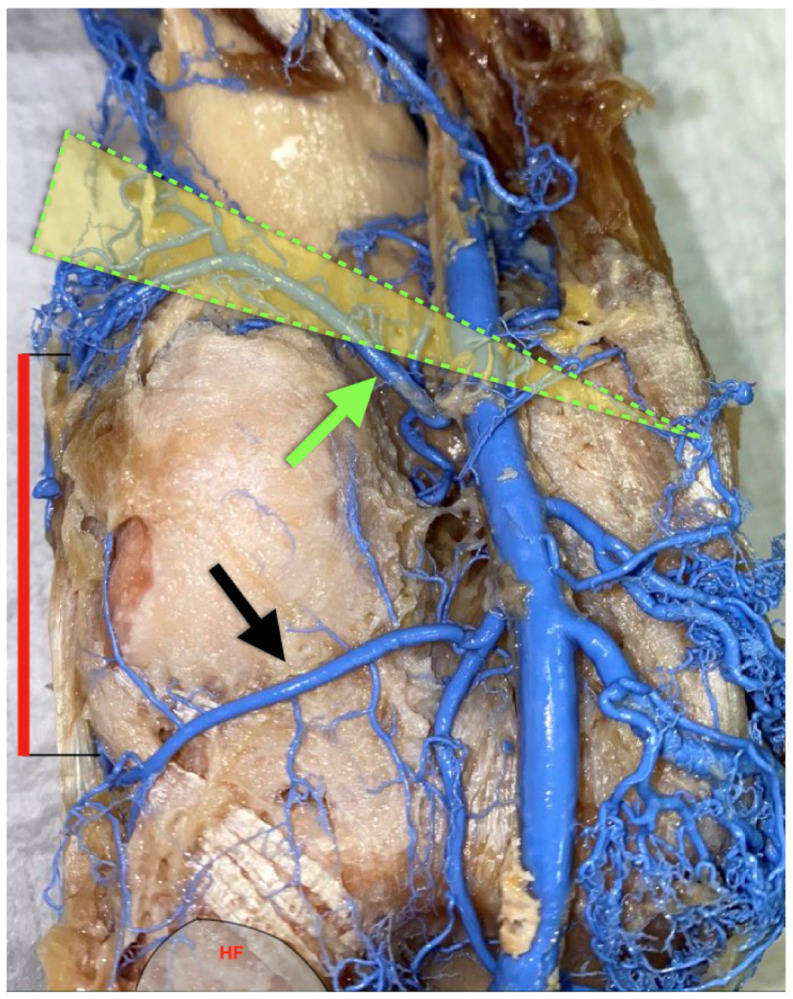
Left knee, posterior view. Mean distance of superior lateral genicular artery (green arrow) from joint line at the lateral knee (red line) was measured to be 52.2 mm ± 6.2 mm by Barner et al. [3]. Bissichia et al. [4] found that the superior lateral genicular artery was in a risk zone during opening-wedge distal femoral osteotomy (green triangle). The median distance from the osteotomy site approximated 3 mm. Black arrow: inferior lateral genicular artery; HF: head of fibula.

**Figure 5:**
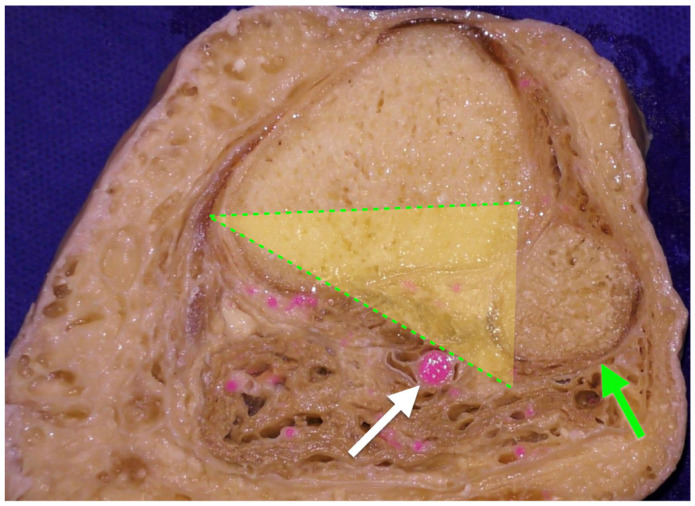
Axial cut of the right tibia approximately at 3 cm below the joint line. The yellow triangle represents a safe zone during medial open wedge high tibial osteotomy in the axial plane in order to avoid popliteal artery damage. Safe angle values in the axial plane for medial open wedge high tibial osteotomy were calculated to be 38.9° ± 6.5° (yellow triangle) by Choi et al. [5], assuming that the anterior portion of the safe angle is parallel to the coronal plane and starting at the most medial edge of the tibia (there was no significant difference in these values between flexion and extension). One should keep in mind the inclination of the osteotomy plane (from the medial side of the tibia 3–3.5 cm below the joint line and aiming upwards and towards the fibula head). White arrow: popliteal artery. Green arrow: head of fibula.

**Figure 6:**
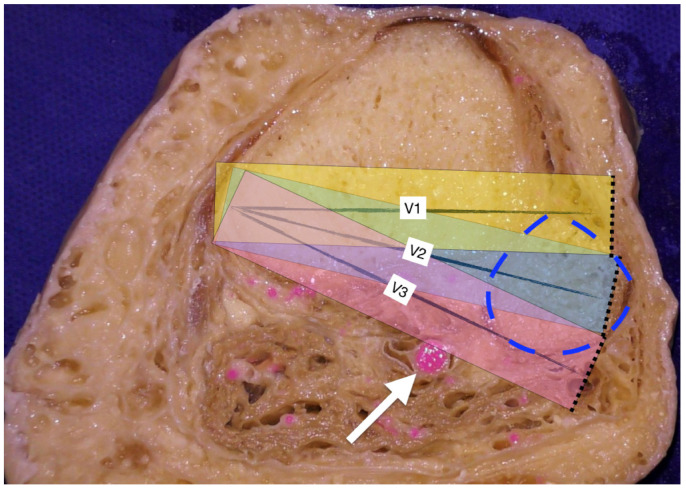
Axial cut of the right tibia approximately at 3 cm below the joint line. Kang et al. measured safe angles and distances in a virtual environment during HTO by taking into consideration that the functioning saw oscillates within a width of 35 mm [6]. They also assumed that the surgeon will perform the osteotomy in three possible virtual directions (V1 = 10^0^ anterior to the anterior edge of the fibula head, V2 = the mid-portion of the fibula head and V3 = 10^0^ posterior to the posterior edge of the fibula head). The width of each parallelogram represents the working width of the oscillating saw. It was assumed that the center of the saw blade runs along each virtual direction (V1-3). There was no risk of popliteal artery injury when sawing was performed along V1. When virtual sawing was performed along V2, Kang et al. found that the popliteal artery was at risk of injury in about 42% of cases. The risk of popliteal artery injury rose to 100% when sawing was performed along V3 [6]. White arrow: popliteal artery. Blue dotted line: head of fibula.

**Figure 7:**
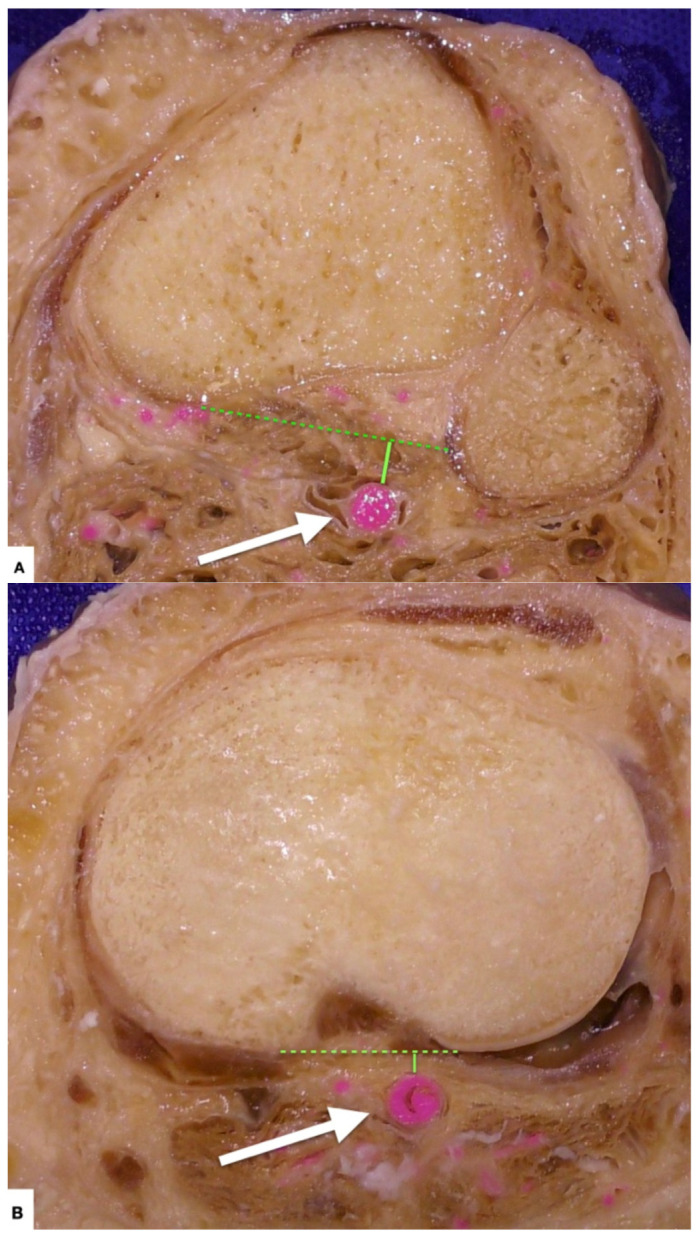
(**A**) Axial view of the tibia at 3 cm below the joint line. Lee et al. found that at the early portion of osteotomy measured at 3 cm below the joint line, the distance (solid green line) between the posterior edge of the proximal tibiofibular joint (green dotted line) and popliteal artery (white arrow) was between 13 and 14 mm [7]. (**B**) Axial view of the tibia just below the tibial plateau. Compared to (**A**), the distance (solid green line) from the tibial artery (white arrow) was shorter (approximately 5 mm) at the end portion of the osteotomy and around the tibia plateau (green dotted line) [7]. The average diameter of the popliteal artery is 8 mm at the subcondylar plane [8].

**Figure 8:**
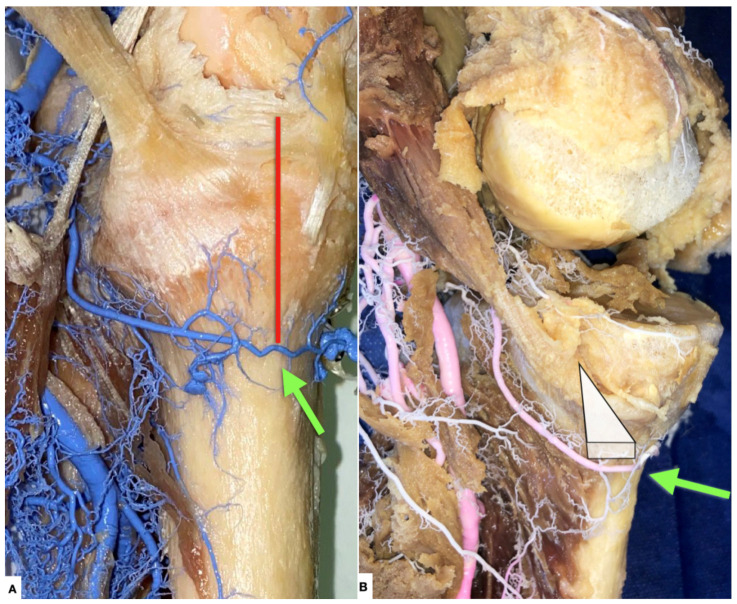
(**A**) Left knee, medial view. Green arrow: inferior medial geniculate artery (IMGA). Red line: distance of IMGA from joint line. Sinno et al. calculated the distance between IMGA and the joint line to be 30 ± 4.3 mm on the sagittal plane and 29.9 ± 4 mm on the coronal plane [9]. There was no statistically significant difference between these two distances. This translates that the IMGA travels below the medial tibial condyle in an almost parallel fashion consistently. Barner et al. calculated the distance between the IMGA and the joint line at the medial knee to be 33 mm ± 6.9 mm [3]. (**B**) Left knee, medial view. Green arrow: inferior medial geniculate artery (IMGA). Bissichia et al. found the IMGA to be at risk during opening-wedge proximal tibial osteotomy, with a median distance from the osteotomy site around 2.1 mm [4]. White triangular wedge-shaped drawing: represents orientation of medial open wedge high tibial osteotomy.

**Figure 9:**
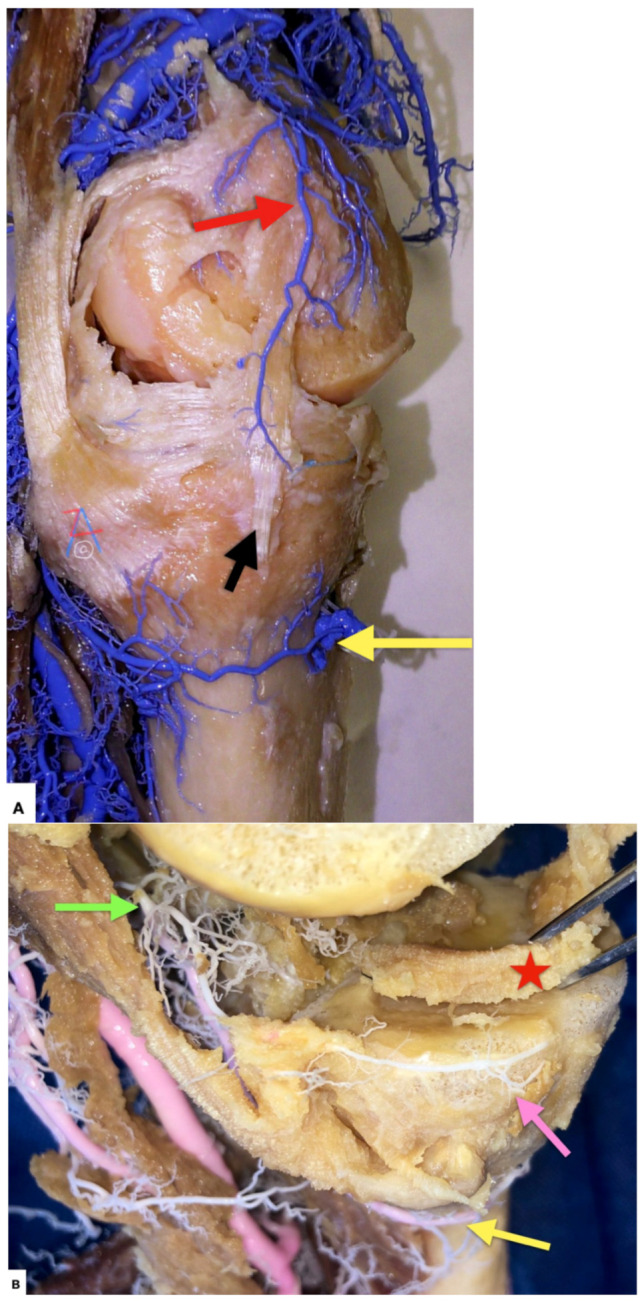
(**A**) Left knee, medial side. The vascular supply of the medial meniscus arises mainly from the medial inferior (yellow arrow in (**B**)) and middle geniculate (green arrow in (**B**)) arteries. From these vessels, a perimeniscal circular vascular ring arises (pink arrow in (**B**)). This peripheral ring of vessels supplies the entire meniscal ligamentous complex and sends branches radially into the meniscal substance. Anastomotic branches towards the circular vascular ring are also supplied by the medial descending geniculate artery (red arrow). The middle peripheral portion of the medial meniscus has a vascular anastomosis through the medial collateral ligament (black arrow) which is closely attached to the meniscus at the joint line, similar to the observation made by Shim et al. [10]. (**B**) Left knee, medial side. The vascular supply (perimeniscal ring depicted with the pink arrow) of the medial meniscus (red star) is depicted as created by the anastomosis of the medial inferior (yellow arrow) and middle geniculate artery (green arrow) The fibrous portion of the menisci located adjacent to the capsule has a rich blood supply. The more central fibrocartilagenous portions are avascular and nourished entirely by synovial fluid.

**Figure 10:**
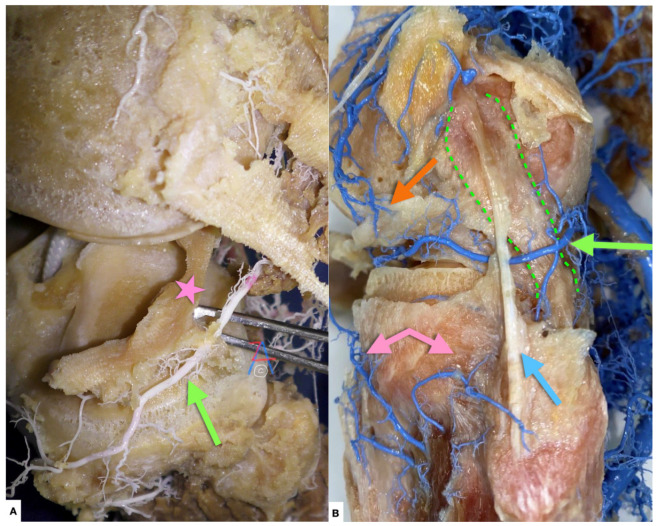
(**A**) Left knee, lateral view. Green arrow: lateral inferior genicular artery, pink star: lateral meniscus. (**B**) The lateral inferior genicular artery (green arrow) provides the blood supply of the peripheral portion of the lateral meniscus, with anastomoses from final branches of the lateral superior (orange arrow) geniculate artery and recurrent branches from the anterior tibial artery (pink arrows). The anterior and posterior horns of the lateral menisci appear more vascular than the bodies of the menisci, as documented by Day et al. [11]. The popliteus tendon (green dotted line) intervenes posteriorly between the lateral meniscus and the inferior lateral genicular artery. This might contribute to a relative avascular zone in the area of the lateral meniscus adjacent to the popliteus tendon, as observed by Day et al. [11]. Light blue arrow: lateral collateral ligament.

**Figure 11:**
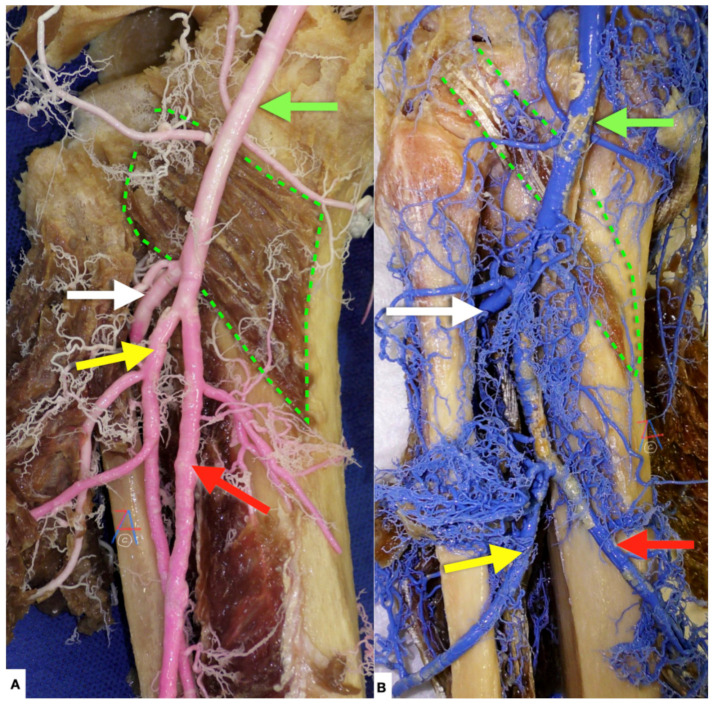
(**A**) Left knee, posterior view. Green arrow: popliteal artery, white arrow: anterior tibial artery, yellow arrow: peroneal artery, red arrow: posterior tibial artery, green dotted line: popliteus muscle. (**B**) Left knee, posterior view. Green arrow: popliteal artery, white arrow: anterior tibial artery, yellow arrow: peroneal artery, red arrow: posterior tibial artery, green dotted line: popliteus muscle. Both images depict the commonest configuration of popliteal artery division. The popliteal artery is divided below the knee into the anterior tibial artery and a common trunk for the posterior tibial and peroneal arteries, found in 92.6% of cases according to Tomaszweski et al. [8] and in 86.49% of cases according to Lambrecht et al. [12].
